# Ribociclib-induced liver injury: a case report

**DOI:** 10.3389/fonc.2023.1256783

**Published:** 2023-12-01

**Authors:** Sofia Schaeffer, Christian Lutz, Michael Dobbie, Luigi M. Terracciano, Matthias Matter, Jürg Vosbeck, Markus H. Heim, Christine Bernsmeier

**Affiliations:** ^1^University Centre for Gastrointestinal and Liver Diseases, Basel, Switzerland; ^2^Department of Oncology, Hôpital du Jura, Delémont, Switzerland; ^3^Department of Biomedical Sciences, Humanitas University, Pieve Emanuele, Milan, Italy; ^4^Department of Pathology, IRCCS Humanitas Research Hospital, Rozzano, Milan, Italy; ^5^Institute of Medical Genetics and Pathology, University Hospital Basel, Basel, Switzerland

**Keywords:** CDK 4/6 inhibitor, ribociclib, hepatotoxicity, DILI, liver necrosis, Hormone-positive HER2-negative breast cancer

## Abstract

**Background:**

Idiosyncratic drug-induced liver injury (DILI) is a rare, unpredictable hepatic adverse event and the most common cause of acute liver failure in Europe and the US. Ribociclib is a potent Cyclin-dependent kinase 4 and 6 (CDK4/6)-inhibitor administered for advanced hormone-receptor (HR)-positive, human epidermal growth factor receptor 2 (HER2)-negative breast cancer. Previous reports have shown hepatotoxicity without liver necrosis related to ribociclib.

**Case presentation:**

A 41-year-old female patient with primary metastatic HR-positive, HER2-negative breast cancer developed liver enzyme elevation under treatment with ribociclib. Ribociclib was withdrawn 8 weeks after initiation due to liver enzyme elevation. A liver biopsy, performed due to further enzyme increase (peak ALT 2836 U/l), onset of jaundice (peak bilirubin 353 µmol/l) and coagulopathy (INR 1.8) two weeks later, revealed acute hepatitis with 30% parenchymal necrosis. Roussel Uclaf Causality Assessment Method (RUCAM) score was 7 points (probable). Under treatment with prednisone (60mg), initiated 2 weeks after drug withdrawal, and subsequently N-acetylcysteine (Prescott regimen) liver enzymes normalized within 8 weeks along with prednisone tapering.

**Conclusion:**

This case illustrates the development of a severe idiosyncratic hepatocellular pattern DILI grade 3 (International DILI Expert Working Group) induced by ribociclib. Routine liver enzyme testing during therapy, immediate hepatologic work-up and treatment interruption in case of liver enzyme elevation are highly recommended. Corticosteroid treatment should be considered in cases of severe necroinflammation.

## Introduction

Approximately 70% of metastatic breast cancers are hormone receptor-positive (HR+), human epidermal growth factor receptor-2-negative (HER-) tumours. As an incurable condition, goal of treatment is to delay tumour progression preferably using anticancer agents with low toxicity. Early standard treatment of HR+ HER2- breast cancers consist of anti-hormonal agents. However, endocrine resistance develops over time and makes cytotoxic chemotherapy necessary (1). Introduction of a novel drug class, the cyclin-dependent kinase (CDK) 4/6 inhibitors, has resulted in relevant improvement in overall survival. Therefore, CDK 4/6 inhibitors are now approved as first- and second-line treatment in the setting of advanced HR+ HER2-negative breast cancer (2–4). Currently, there are three commercially available oral selective CDK 4/6 inhibitors (ribociclib, palbociclib and abemaciclib).

Idiosyncratic drug-induced liver injury (DILI) describes rare, unpredictable damage that commonly-used drugs cause to the liver occurring with variable latency to drug exposure. In contrast to the intrinsic (or direct) type of liver toxicity, which is dose-related and arises shortly after drug exposure in most individuals, idiosyncratic DILI is determined by the interaction of host and environmental factors with the causative drug, even when taken in the recommended dosage. Reactive drug metabolites can induce oxidative and organelle stress and interfere with bile acid transport resulting in adaptive immune responses in genetically susceptible individuals. Increasing age, female sex, concomitant alcohol consumption, and metabolic syndrome have been identified as risk factors for DILI (5). DILI is the most frequent cause of acute liver failure in Europe and the US (6).

The definition and diagnosis of DILI are based on a detailed clinical history, biochemical and radiological tests with careful exclusion of alternative causes (7). Based on the first set of laboratory tests available in relation to the clinical event, DILI pattern is classified as hepatocellular, cholestatic, or mixed (5). Liver biopsy is not mandatory for the diagnosis of DILI, yet it can confirm the diagnosis and biochemical classification, and exclude alternative diagnoses. Clinical severity of DILI, categorized into 4 grades, is based on the highest measured biochemical parameters during the course of DILI (7). Given specific tests to confirm DILI are lacking, causality assessment tools are warranted and the most commonly used diagnostic tool for DILI is the Council for International Organizations of Medical Sciences (CIOMS) scale, also referred to as the Roussel Uclaf Causality Assessment Method (RUCAM). The scale is composed of seven different criteria and categorizes DILI as “definite”, “highly probable”, “probable”, “possible”, unlikely” or “excluded” (5).

Treatment of DILI consists of immediate withdrawal of the offending drug, supportive measures and timely referral to a liver transplantation centre in case of imminent acute liver failure. Despite limited evidence for benefits, corticosteroids may be empirically administered in cases of histologically verified necroinflammation.

Previous case reports have illustrated grade 4 hepatotoxicity (8) and fulminant hepatitis (9) due to ribociclib. This case report demonstrates the first case of ribociclib-related histologically confirmed DILI with extensive liver necrosis.

## Case presentation

A 41-year-old Caucasian woman was diagnosed with HR+ HER2- breast cancer with bone, pulmonary, and pleural metastases. Treatment with gosereline (GnRH analogue) every 12 weeks, letrozole (2.5mg daily) and ribociclib was initiated simultaneously. Ribociclib 600mg was administered in 4-week cycles (21/7-day schedule). Before initiation of ribociclib the patient had normal liver enzymes and there were no signs of liver metastases on radiological examination. There was no history of chronic liver disease, alcohol or drug abuse and the patient did not take any other medications.

After one cycle of ribociclib a significant reduction of the tumour load was confirmed radiologically. After the second cycle of ribociclib, a relevant elevation of transaminases (AST 252 U/l, ALT 639 U/l, ALP and bilirubin within normal range) was observed with consecutive withdrawal of ribociclib and letrozole. Investigations for acute viral hepatitis (Hepatitis A, B, C, E, HSV1/2, CMV, EBV, VZV, HHV6 and HHV7) and autoimmune liver disease (anti-nuclear antibody, anti-smooth muscle antibody, anti-liver-kidney-microsomal type 1 antibody, level of immunoglobulin IgG, anti-neutrophil antibody, anti-proteinase 3 antibody and anti-myeloperoxidase antibody) were negative or within normal range, respectively. Ultrasound of the liver did not show any abnormalities.

**Figure 1:**
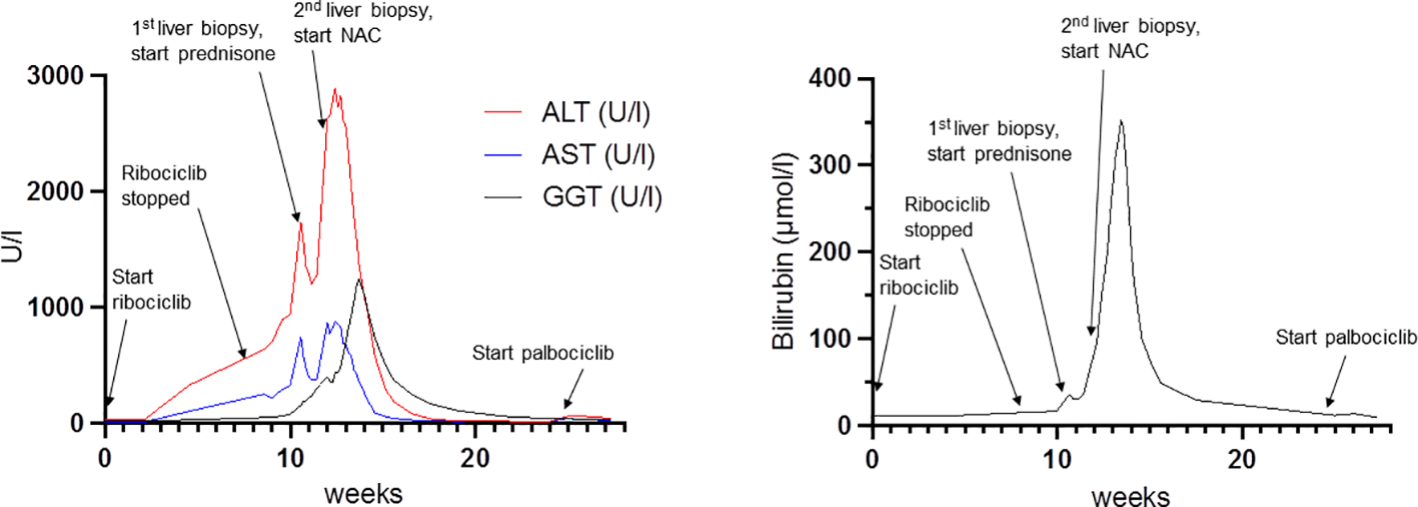
Course of liver enzyme elevation in relation to ribociclib administration and predisone, N-acetylcysteine treatment. NAC: N-Acetylcysteine.

**Figure 2:**
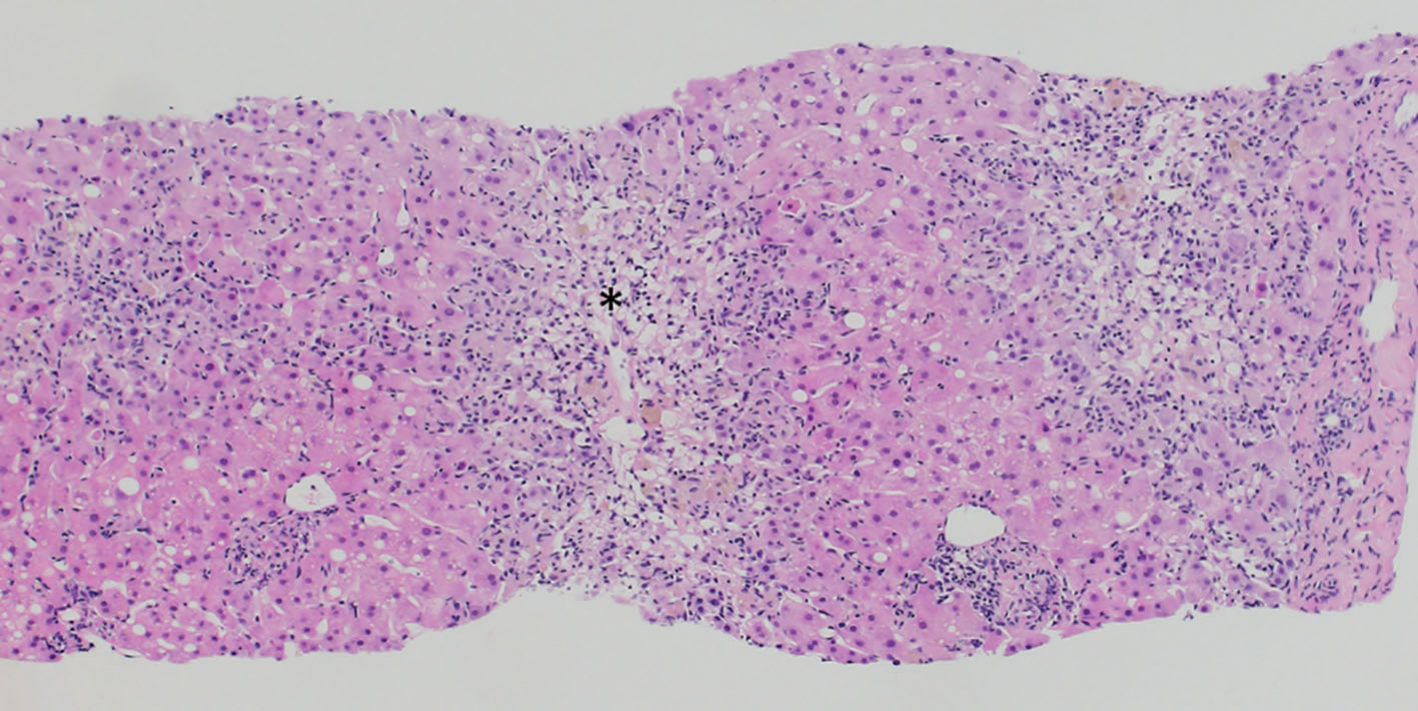
Liver biopsy result demonstrating extensive centrilobular necrosis (asterisk) with numerous apoptotic cells, moderate lymphohistiocytic portal and lobular inflammation and mild cholestasis. (Haematoxylin Eosin stain).

**Figure 3:**
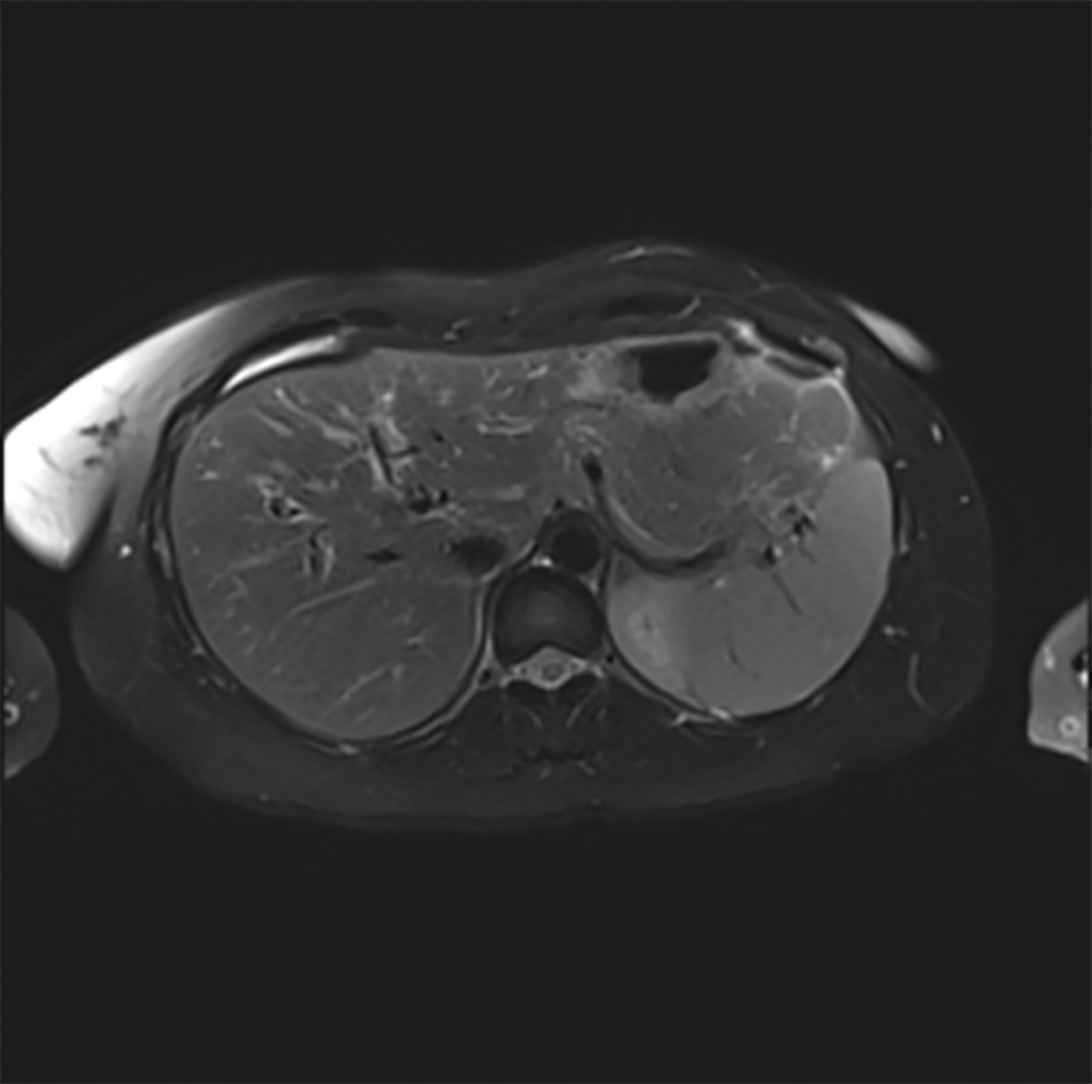
T2-weighted MRT scan of the liver (transverse section) showing normal liver parenchyma and normal biliary ducts.

Despite discontinuation of ribociclib, liver enzymes continued to rise (**Figure 1**). Acute DILI with hepatocellular pattern was suspected and RUCAM score was 7 points supporting a probable causal association of liver injury due to ribociclib therapy. Liver biopsy two weeks after discontinuation of ribociclib showed extensive lobular necrosis accentuated in zone III (30% of liver parenchyma) with mild portal und moderate lobular lymphohistiocytic inflammation. The histology did not show evidence for metastasis or viral hepatitis. In accordance to previous case reports, treatment with prednisone 60mg OD was initiated 14 days after treatment discontinuation (8, 9). Due to further increase in liver enzymes (peak ALT 2896 U/l, peak AST 875 U/l), onset of jaundice (peak bilirubin 353 µmol/l), coagulopathy (peak INR 1.8), and decrease in serum albumin levels (nadir 28g/l) despite corticosteroid treatment, a second liver biopsy was performed 25 days after ribociclib withdrawal. Results showed a similar result with 30% lobular necrosis accentuated in zone III with numerous ceroid-containing macrophages and surrounded by apoptotic cells, moderate lymphohistiocytic portal and lobular inflammation and mild cholestasis. Moreover, there were signs of endothelialitis and slight siderosis but no evidence for fibrosis (**Figure 2**). Again, histology suggested no alternative causes for liver injury. Additional investigation with MRI excluded other parenchymal liver and bile duct pathology (**Figure 3**). Due to the extent of liver necrosis, treatment with N-acetylcysteine according to Prescott et al. was added (10). Prednisone was tapered after transaminase had peaked. Liver enzymes and bilirubin normalised within 10 weeks after discontinuation of ribociclib. Encephalopathy was not observed at any time during the course of liver injury.

After liver enzyme normalisation, tumour progression led to reintroduction of letrozole which did not affect liver enzymes. Given previous case series described safety of sequential CDK 4/6 inhibitor re-challenge after liver injury due to ribociclib (8, 11, 12) and as the oncological response to ribociclib was very good, therapy with palbociclib 125mg OD was added 6 weeks after liver enzyme normalization with close laboratory monitoring. Unfortunately, the patient developed mild liver enzyme elevation (ALT peak 64 U/l, ASAT peak 44 U/l) after only 4 subsequent doses of palbociclib. After discontinuation of palbociclib, liver enzymes normalized. Due to tumour progression, treatment with letrozole and GnRH analogue were reinitiated thereafter. No further hepatopathy was observed under this therapy.

The case was reported to the pharamcovigilance unit of the Swiss national authority for therapeutic products (Swissmedic). Written informed consent was obtained from the patient for the publication of this case report.

## Discussion

CDK 4/6 inhibitors interfere with retinoblastoma (Rb) protein phosphorylation and thereby inhibit cell cycle progression and uncontrolled tumour growth (13). Dysregulation in the cyclin D-CDK4/6-pRb pathway is known to be a key mediator of endocrine resistance in HR+ breast cancer (14). Ribociclib is metabolised in the liver by CYP3A4 and is eliminated via the biliary system. The most common side effect of ribociclib is neutropenia. In the MONALEESA-2 phase III trial, grade 3 or 4 abnormal liver function tests were found in 8.4 and 1.8% of patients in the ribociclib plus letrozole vs. 2.4 and 0% in the letrozole plus placebo arm, respectively (15). This resulted in the recommendation for monitoring liver function before and during treatment. Additionally, a strategy on how to proceed in case of hepatic adverse events was provided. A meta-analysis showed an increased risk for hepatic toxicity under ribociclib compared to palbociclib and abemaciclib, respectively (absolute risk for grade 3-4 ALT increase for ribociclib 0.097, for palbociclib 0.034, for abemaciclib 0.046, respectively) (16).

Previous case reports on hepatic injury following ribociclib treatment are listed in **Table 1** (8, 9, 17). Similarly, all previous cases had hepatocellular pattern of liver injury and further increase in transaminases was observed for several weeks despite immediate discontinuation of ribociclib after abnormality was noticed. None of the events were fatal and mild encephalopathy (grade 1) was observed in only one case (9). Liver biopsy, performed in a single case, demonstrated fulminant hepatitis but no necrosis (9). Steroid treatment was initiated in 3 of 4 cases and seemed to accelerate liver enzyme normalisation compared to the case not treated with steroids. In contrast to the previous case reports, liver enzymes did not immediately decrease after steroid initiation in our case. Rather, further increase in transaminases for another 2 weeks despite an initial dose of 60 mg prednisone (1 mg/kg body weight) daily was observed. In our case, the rationale for starting steroid treatment was supported by the presence of extensive liver necrosis with concurrent inflammation. We assume that the anti-inflammatory effects under steroid treatment caused a deceleration of the inflammatory and immunologic reactions and therefore prevented the development of an overwhelming and possibly fatal acute liver failure. The rationale for adding N-acetylcysteine was due to the lack of improvement in histological liver regeneration and liver enzyme elevation despite limited evidence in non-acetaminophen-induced DILI without diagnostic criteria for acute liver failure (18). In our opinion, there is no interference with the co-medication letrozole as the latter has only been attributed to mild and transient liver injury and is not deemed to cause drug interactions. To our knowledge, this is the first report of a case of acute, extensive liver necrosis due to ribociclib intake.

**Table 1:** Comparison of previous case reports on ribociclib-induced liver injury.

	Sex, age, ethnicity	Tumor characteristics	Primary tumour treatment	Ribociclib start and dosage	latency of DILI onset after starting ribociclib	DILI pattern, clinical features of DILI	Peak liver function tests (days after ribociclib discontinuation)	Liver biopsy	Treatment of DILI	Normalization of liver enzymes (days after ribociclib withdrawal/starting corticosteroids)	Rechallenge with CDK4/6-inhibitor
**Finnsdottir S. et al. (2021) (** 8)	Female, 54, Caucasian	ER+, HER2- with bone metastases	Letrozole (2.5mg daily), 8x Epirubicn and cyclophosphamide	6 months after initial diagnosis, 600mg per day	1 cycle (21d)	Hepatocellular, pruritus and jaundice	AST ~1300 U/l (~day 31) ALT ~1160 U/l (~day 21)	no	Ribociclib withdrawal, prednisolone 40mg/d with tapering	Yes (~2 months/1 month)	Palbociclib 125mg per day, successful
	Female, 66, Caucasian	ER+, HER2- with secondary bone and lung metastases >10 years after initial diagnosis	Partial mastectomy, axillary lymph node dissection and adjuvant radiotherapy with temporary anastrozole	12 years after initial diagnosis, 600mg per day	3 cycles	Hepatocellular, nausea and fatigue	AST ~930 U/l (~day 15) ALT 1112 U/l (~day 35)	no	Ribociclib withdrawal, prednisolone 40mg/d with tapering	Yes (~11 weeks/5 weeks)	Palbociclib 125mg per day, successful
**Topcu A. et al. (2022) (** 9)	Female, 54,?	HR+, HER2 score 2 with primary lung and bone metastases	Trastuzumab/Docetaxel and radiotherapy, maintenance with anastrozole and trastuzumab	3 years after initial diagnosis, 600mg per day	2 cycles	Hepatocellular, hepatic encephalopathy Grade 1	AST ~1300 U/l (~day 56) ALT ~750 U/l (~day 56)	Fulminant toxic hepatitis	Ribociclib withdrawal, prednisolone 1mg/kg	Yes (11 weeks/3 weeks)	No
**Meynard L. et al. (2020) (** 17)	Female, 59,?	HR+, HER2- with secondary disseminated bone metastases (17 years after initial diagnosis)	Lumpectomy and radiotherapy	17 years after initial diagnosis, 600mg per day	4 cycles	Hepatocellular, asymptomatic	AST ~ 300 U/l (day 32) ALT ~510 U/l (day 32)	no	Ribociclib withdrawal	Yes (4 months/-)	Palbociclib 75-100 mg per day, successful

Interestingly, previous case reports described successful rechallenge with another CDK4/6-inhibitor (palbociclib of abemaciclib) with no further hepatic injury (8, 9, 15, 19). In our patient however, palbociclib was not tolerated causing again mild liver enzyme elevation. Pre-marketing clinical trials of CDK4/6-inhibitors indicated a drug-related liver toxicity rather than a class effect justifying a rechallenge (20). This assumption was, however, not supported in our case. Despite only minimal liver enzyme elevation under palbociclib, further continuation of palbociclib was not supported given the significant extent of previous liver necrosis and the risk of further hepatic injury. Unfortunately, in this scenario treatment of metastatic breast cancer was limited to endocrine therapy.

The exact mechanism of ribociclib-induced liver injury with extensive liver necrosis remains unclear. We hypothesize a multifactorial pathogenesis with reactive metabolite formation as suggested by Raschi et al. (20) and a significant immunologic reaction as supported by the MONALEESA-2 trial where several cases of DILI with autoimmune-like pattern were observed (15). Moreover, ribociclib is a recognised inhibitor of hepatic transporters, especially bile salt export pump (BSEP). Ribociclib was shown to cause dual inhibition of BSEP and basolateral efflux systems, which are additional susceptibility factors for cholestasis which might explain the significant jaundice observed in our patient (20). Additionally, genetic and perhaps also hormonal factors might play a role in the pathogenesis of ribociclib-induced DILI.

In conclusion, this case supports the need for close drug monitoring under treatment with CDK4/6-inhibitors. Prompt hepatologic work-up is crucial for a timely diagnosis and treatment initiation. Generally, easily accessible reporting systems are necessary to ensure drug safety.

## Data availability statement

The original contributions presented in the study are included in the article/supplementary material. Further inquiries can be directed to the corresponding author.

## Ethics statement

Written informed consent was obtained from the individual(s) for the publication of any potentially identifiable images or data included in this article.

## Author contributions

SS: Conceptualization, Data curation, Visualization, Writing – original draft, Writing – review & editing, Formal Analysis, Investigation. CL: Data curation, Writing – review & editing. MD: Data curation, Supervision, Validation, Writing – review & editing. LT: Formal Analysis, Investigation, Writing – review & editing. MM: Formal Analysis, Investigation, Writing – review & editing. JV: Formal Analysis, Investigation, Writing – review & editing. MH: Investigation, Supervision, Writing – review & editing. CB: Conceptualization, Investigation, Supervision, Validation, Writing – review & editing.
